# Investigation of adolescent cases diagnosed with idiopathic/genetic generalized epilepsy in terms of self-esteem, aggression, body perception, and alexithymia

**DOI:** 10.1007/s00431-026-06855-0

**Published:** 2026-03-19

**Authors:** Sema Bozkaya-Yilmaz, Safa Mete Dagdas, Emre Firat, Gunce Basarir, Gonca Ozyurt, Nihal Olgac-Dundar, Pinar Gencpinar

**Affiliations:** 1 Department of Pediatric Neurology, Bursa State Hospital, Bursa, Turkey; 2 Department of Pediatric Neurology, Izmir State Hospital, Izmir, Turkey; 3 Department of Pediatric Allergy and Immunology, Dr. Behcet Uz Hospital, Izmir, Turkey; 4 Department of Pediatric Neurology, Haseki Training and Research Hospital, Istanbul, Turkey; 5 Department of Child and Adolescent Psychiatry, Faculty of Medicine, Izmir Katip Çelebi University, Izmir, Turkey; 6 Department of Pediatric Neurology, Faculty of Medicine, Izmir Katip Çelebi University, Izmir, Turkey

**Keywords:** Adolescent, Epilepsy, Self-esteem, Aggression, Body perception, Alexithymia

## Abstract

This study examined self-esteem, body image, alexithymia, and aggression in adolescents with idiopathic/genetic generalized epilepsy (IGE). These domains were selected because they may be affected not only by epilepsy itself but also by stigma, difficulties in emotional regulation, or treatment-related effects. Assessing these domains together was intended to examine whether IGE might be associated with additional psychosocial strain in otherwise clinically stable adolescents. We carried out a case–control study including 45 adolescents with IGE and 45 controls matched for age and sex. Controls were selected from outpatient services and were required to have no neurological disorders. Psychiatric disorders were assessed using the K-SADS (Kiddie Schedule for Affective Disorders and Schizophrenia) interview; no clinically significant psychiatric diagnoses were identified in the control group. All participants completed standardized Turkish questionnaires on the same day.Within the epilepsy group, 20.0% were diagnosed with juvenile absence epilepsy (JAE), 22.2% with juvenile myoclonic epilepsy (JME), and 57.8% with epilepsy with generalized tonic-clonic seizures alone (EGTCSA). Across all four psychological measures, the epilepsy and control groups showed no statistically significant differences (all p > 0.05). No significant differences were found in subgroup analyses based on epilepsy syndrome or seizure frequency. *Conclusion*: Adolescents with well-controlled IGE and minimal psychiatric comorbidities demonstrated comparable levels of psychosocial functioning to their healthy peers. This finding is consistent with evidence suggesting that psychosocial difficulties in epilepsy are often linked to comorbidity and contextual factors rather than to the diagnosis alone. Studies conducted in broader settings and with larger, more varied samples will be needed to better understand how medical, emotional, and environmental factors jointly influence psychosocial outcomes in this population.

**Table:** 

What is known: • Adolescents with epilepsy are frequently considered at increased risk for psychosocial difficulties, including lower self-esteem, emotional instability, and body image concerns.		
What is new: • In this study, no significant differences were found in self-esteem, body image, alexithymia, or aggression among Turkish adolescents with idiopathic/genetic generalized epilepsy compared with healthy controls. These findings suggest that, when seizures are well controlled and psychiatric comorbidities are limited, epilepsy itself may not confer additional psychosocial vulnerability.

What is known:

• Adolescents with epilepsy are frequently considered at increased risk for psychosocial difficulties, including lower self-esteem, emotional instability, and body image concerns.

What is new:

• In this study, no significant differences were found in self-esteem, body image, alexithymia, or aggression among Turkish adolescents with idiopathic/genetic generalized epilepsy compared with healthy controls. These findings suggest that, when seizures are well controlled and psychiatric comorbidities are limited, epilepsy itself may not confer additional psychosocial vulnerability.

## Introduction

Epilepsy is the most common chronic neurological condition affecting children and adolescents in the world, with idiopathic/genetic generalized epilepsies (IGE) representing a major proportion [1]. IGE is defined by characteristic clinical and electroencephalographic patterns; however, increasing attention has been directed toward its broader cognitive, behavioral, and psychosocial implications [2]. These consequences typically extend beyond the seizures themselves and may shape multiple aspects of a young person’s daily life.

Adolescence is a phase of escalation in social expectations and a process of identity formation, a time when young people also tend to become more aware of how they are viewed by their peers. These typical challenges often overlap with illness-related issues including unpredictable seizures, fear of stigma, side effects of medicines, and limits on social involvement. Recent studies have shown that adolescents with epilepsy tend to note more emotional and behavioral difficulties than their healthy peers, and in some cases even more than adolescents with other chronic medical problems [3, 4]. A long-term cohort study has also shown that negative social outcomes tend to appear particularly among individuals with complicated epilepsy or ongoing seizures, whereas outcomes are more favorable in uncomplicated cases [5]. These observations emphasize the need to identify the specific psychological elements in which adolescents with epilepsy may be particularly vulnerable.

In this context, special attention may need to be given to certain psychological dimensions in adolescents with epilepsy. Self-esteem is closely related to perceived autonomy and peer acceptance; these relationships can be disrupted by stigma and restrictions associated with epilepsy [6]. Aggression can arise from frustration, emotion regulation disorders, or the accumulated burden of chronic illness [7]. Concerns about body image may be influenced by drug-induced changes in appearance and increased sensitivity to physical perceptions during adolescence [8]. Alexithymia, characterized by difficulty recognizing and expressing emotions, has been associated with various neurological disorders and may play a role in maladaptive coping or interpersonal difficulties in youth with epilepsy [9]. Considering these domains simultaneously may help describe psychosocial functioning in adolescents with IGE more comprehensively. On this basis, the present study evaluated self-esteem, aggression, body image, and alexithymia in adolescents with IGE.

## Methods

This study was carried out at İzmir Tepecik Training and Research Hospital’s Department of Child Neurology. A total of 90 adolescents were included: 45 diagnosed with idiopathic/genetic generalized epilepsy (IGE) and 45 age- and sex-matched healthy controls. Clinical and demographic data were collected for all participants.

### Study design and procedure

This study was conducted as a case–control investigation comparing adolescents with IGE to age- and sex-matched healthy controls. All assessments were conducted at a single time point during a routine clinical visit. Standardized instructions were given to participants prior to the completion of the psychological measures, and all questionnaires were self-administered with a trained researcher guiding the assessment process. The full assessment session for each individual took approximately one hour.

All adolescents, both patients and controls, underwent a psychiatric assessment by the same child psychiatrist using the Kiddie Schedule for Affective Disorders and Schizophrenia (K-SADS) to ensure developmental appropriateness and to identify psychiatric comorbidities.

The study was conducted between October 2020 and June 2021. No follow-up period was included; therefore, the study does not represent a prospective cohort design. Ethical approval was obtained from the Clinical Research Ethics Committee of the Health Sciences University, Tepecik Training and Research Hospital (Decision No: 02, dated 08/10/2020). Written informed consent was obtained from all adolescents and from their parents or legal guardians prior to participation.

### Patient group

Patients with idiopathic/genetic generalized epilepsy were consecutively recruited from the Department of Child Neurology. Inclusion criteria were as follows:

- A confirmed epilepsy diagnosis according to ILAE criteria
- Age 12–18 years
- Normal cranial MRI
- Normal motor and mental development, as determined by neurological follow-up and the K-SADS interview

Minimal psychiatric comorbidities (ADHD, depressive disorders, or anxiety disorders) were permitted. All other psychiatric disorders resulted in exclusion.

Additional exclusion criteria included incomplete data, the presence of any neurodevelopmental disorder, chronic medical illness requiring regular medication, or abnormal MRI findings.

Although all patients were diagnosed prior to the most recent ILAE updates, epilepsy syndromes were reclassified according to current ILAE terminology based on documented electroclinical features.

### Control group

Healthy controls were recruited from the general pediatric outpatient clinic among adolescents presenting for non-neurological and non-psychiatric reasons. Inclusion criteria were as follows:

- Age 12–18 years
- Absence of any neurological or neurodevelopmental disorder
- Age-appropriate development was confirmed through the K-SADS evaluation, and there was no chronic medical condition or regular medication use

Exclusion criteria mirrored those applied to the patient group. Psychiatric disorders were assessed using the K-SADS interview. Although minimal psychiatric comorbidities (ADHD, depressive disorders, or anxiety disorders) were not predefined exclusion criteria, no such diagnoses were identified in the control group. Adolescents using chronic medication or those with incomplete data were excluded. Controls were matched to the epilepsy group by age and sex.

### Clinical and baseline variables

Demographic data (age and sex) and the presence of minimal psychiatric comorbidities (ADHD, depressive disorder, or anxiety disorders) were collected in all patients.

Furthermore, other clinical correlates were collected from adolescents with epilepsy to identify the status of the disease. These included the following:

- Epilepsy classification according to ILAE criteria
- Current antiseizure medication (ASM)
- Total number of ASMs (monotherapy vs. polytherapy)
- Treatment response and presence and nature of ASM-related adverse effects

Quantitative distributions for these variables are reported in the “Results” section.

### Scales

#### Buss-Perry Aggression Questionnaire (BPAQ)

It was assessed using the Buss–Perry Aggression Questionnaire, a 34-item measure rated on a five-point Likert scale, covering physical aggression, verbal aggression, anger, and hostility. Higher scores on the scale indicate greater levels of aggressive thoughts and behaviors. The Turkish adaptation of the scale has demonstrated adequate validity and reliability in adolescent samples [10–13].

#### Body Perception Scale

It was measured using the 40-item Turkish adaptation of the Body Perception Scale, scored on a five-point Likert format. Higher scores on this scale reflect a more positive evaluation of one’s body. The Turkish version has been validated and shown to be reliable for use in adolescent populations [14].

#### Alexithymia Scale for Children (CAS)

It was evaluated with the 20-item Alexithymia Scale for Children, scored on a three-point Likert scale, comprising three subdimensions: difficulty identifying feelings, difficulty describing feelings, and externally oriented thinking. Higher scores on this scale indicate greater levels of alexithymic features. Its Turkish adaptation has established validity and reliability for children and adolescents [15].

#### Self-Esteem Scale

It was measured using the 10-item Self-Esteem subscale of the Rosenberg Self-Esteem Scale, rated on a four-point Likert scale. Higher scores on this scale indicate greater perceived self-esteem. The Turkish adaptation has been validated and is widely used in adolescent research [16].

### Statistical analysis

Data were analyzed using IBM SPSS Statistics version 21. Continuous variables were described with means, standard deviations, and medians as appropriate. Examination of normality through both graphical inspection and formal tests suggested that the distributions deviated from normal.

Given this distribution pattern, non-parametric methods were preferred. Differences between the epilepsy and control groups were examined using the Mann–Whitney *U* test. When comparisons involved more than two categories, the Kruskal–Wallis test was applied. Categorical variables were evaluated using chi-square analysis, with Fisher’s exact test used when cell counts were small. Statistical significance was defined as *p* < 0.05.

A priori power calculation was not performed. As a result, the sample size may not have been sufficient to detect small differences between groups. In addition, multivariable regression modeling was not undertaken because of both sample size considerations and the distributional properties of the data; this should be taken into account when interpreting the findings.

## Results

### Sample characteristics

A total of 90 adolescents were included in the study. Age and sex distributions were similar between the IGE group and control group (Table 1).

**Table 1:** Baseline characteristics of adolescents in the control group and those with idiopathic/genetic generalized epilepsy (IGE)

Variable	Control group (*n* = 45)	IGE group (*n* = 45)
Age (months), mean ± SD	175.5 ± 22.9	180.0 ± 29.4
Age (months), median (min–max)	180.0 (132–240)	180.0 (132–240)
Sex	*n* (%)	*n* (%)
• Male	21 (46.7%)	21 (46.7%)
• Female	24 (53.3%)	24 (53.3%)

### Clinical characteristics of the IGE group

Detailed clinical features of adolescents with IGE, including epilepsy syndrome classification, EEG findings, seizure frequency, psychiatric comorbidities, and treatment characteristics, are presented in Table 2.

**Table 2:** Clinical characteristics of adolescents with idiopathic/genetic generalized epilepsy (IGE)

		*n*	%
Epilepsy classification	JAE	9	20.0
	JME	10	22.2
	EGTCSA	26	57.8
Comorbid psychiatric disease	None	40	88.9
	Yes	5	11.1
Type of psychiatric disorder (among those with comorbidity)	ADHD	2	40.0
	Depression	1	20.0
	Anxiety disorders	2	40.0
Pre-treatment EEG	Normal	11	24.4
	Abnormal	34	75.6
Pre-treatment EEG findings	Focal	3	8.8
	Generalized	31	91.2
Post-treatment EEG	Normal	35	77.8
	Abnormal	10	22.2
Post-treatment EEG findings	Focal	1	10.0
	Generalized	9	90.0
Seizure frequency	> Once a year	24	53.3
	> Once a month	9	20.0
	> Once a week	4	8.9
	> Once a day	1	2.2
	Single seizure	7	15.6

*JAE* juvenile absence epilepsy, *JME* juvenile myoclonic epilepsy, *EGTCSA* epilepsy with generalized tonic–clonic seizures alone, *ADHD* attention-deficit/hyperactivity disorder

### Comparison between adolescents with IGE and controls

No significant differences were observed between the IGE and control groups in terms of body image, self-esteem, aggression, or alexithymia scores (Table 3).

**Table 3:** Psychological scale scores in adolescents with idiopathic/genetic generalized epilepsy (IGE) and controls

Scale	IGE group (*n* = 45)	Control group (*n* = 45)	*p*-value
Body image, median (min–max)	142.0 (110–193)	146.0 (107–219)	0.445*
Rosenberg Self-Esteem Scale, mean ± SD	27.1 ± 3.6	27.5 ± 3.4	0.611**
Buss–Perry Aggression Questionnaire, median (min–max)	76.0 (43–124)	78.0 (39–132)	0.611*
Alexithymia Scale for Children, mean ± SD	18.6 ± 7.2	16.6 ± 6.6	0.190**

^*^Mann–Whitney *U* test

^**^Independent samples *t*-test

### Comparisons within the IGE group by epilepsy syndrome

Psychological scale scores did not differ significantly between adolescents with epilepsy with generalized tonic–clonic seizures alone (EGTCSA), juvenile myoclonic epilepsy (JME), or juvenile absence epilepsy (JAE) (Table 4).

**Table 4:** Psychological scale scores by epilepsy syndrome in adolescents with idiopathic/genetic generalized epilepsy (IGE)

Scale	EGTCSA (*n* = 26)	JME (*n* = 10)	JAE (*n* = 9)	*p*-value
Body image, median (min–max)	148.0 (110–193)	134.5 (111–176)	132.0 (126–157)	0.079*
Rosenberg Self-Esteem Scale, median (min–max)	27.0 (22–36)	26.5 (22–31)	28.0 (22–36)	0.829*
Buss–Perry Aggression Scale, median (min–max)	79.0 (43–124)	66.5 (59–109)	65.0 (53–94)	0.150*
Alexithymia Scale for Children, median (min–max)	18.0 (5–34)	15.5 (3–33)	20.0 (15–30)	0.470*

^*^Nonparametric test

### Comparisons within the IGE group by seizure frequency

Body image, self-esteem, aggression, and alexithymia scores were also similar across seizure frequency categories (Table 5).

**Table 5:** Psychological scale scores according to seizure frequency in adolescents with idiopathic/genetic generalized epilepsy (IGE)

Scale	> Once per year or single seizure (*n* = 31)	> Once per month (*n* = 9)	> Once per week or daily (*n* = 5)	*p*-value
Body image, median (min–max)	136.0 (111–193)	142.0 (110–190)	157.0 (136–176)	0.263*
Rosenberg Self-Esteem Scale, median (min–max)	28.0 (22–36)	26.0 (22–31)	23.0 (22–36)	0.839*
Buss–Perry Aggression Scale, median (min–max)	79.0 (43–124)	73.0 (53–94)	76.0 (62–102)	0.500*
Alexithymia Scale for Children, median (min–max)	18.0 (5–34)	16.0 (3–30)	20.0 (12–26)	0.857*

^*^Nonparametric test

## Discussion

Epilepsy is a common neurological condition in childhood and adolescence, and its psychosocial implications are not shared across individuals. Although various studies have established challenges associated with self-esteem, body image, emotional regulation, and behavioral functioning in young people with epilepsy, other studies have demonstrated that these challenges are far from consistent [17–20]. In many cases, psychosocial challenges are more associated with co-occurring psychiatric symptoms, adverse effects of antiseizure medications, and environmental or family-related factors, rather than with epilepsy itself [21–23]. These mixed findings show that the psychological functioning of adolescents with epilepsy is likely characterized by interactions between clinical and emotional factors, as well as contextual factors, beyond those factors determined by the diagnosis alone.

Recent large-scale screening and observational studies further emphasize this heterogeneity, demonstrating that clinically significant emotional and behavioral symptoms tend to cluster in subgroups of individuals with higher seizure burden, sleep difficulties, or neurodevelopmental traits, whereas perceived social support may remain relatively preserved across both pediatric and adult epilepsy populations [24]. Such findings support a multidimensional conceptualization of psychosocial vulnerability in epilepsy rather than a uniform risk attributed solely to the diagnosis itself [25].

This study examined psychosocial functioning in adolescents with IGE beyond routine clinical indicators such as seizure control and medication-related effects. Given the multidimensional nature of psychosocial well-being, we assessed self-esteem, alexithymia, body image, and aggressive behaviors in the same cohort. Self-report measures provided insight into adolescents’ subjective experiences alongside clinical evaluation. The study was descriptive and not designed to assess therapeutic change.

The deliberate selection of a relatively homogeneous group of adolescents with well-controlled IGE and minimal psychiatric comorbidities was done to limit clinical heterogeneity and reduce potential confounding factors. By including a clinically stable sample, we aimed to reduce the influence of active psychiatric symptoms or uncontrolled seizures and to better explore whether the IGE diagnosis itself was linked to psychosocial differences.

### Alexithymia

Alexithymia has been reported in some patients with epilepsy, usually in relation to depressive symptoms or other forms of emotional dysregulation [26]. In the present study, however, adolescents with IGE did not differ from their healthy peers in alexithymia scores. This finding is in line with previous observations suggesting that alexithymic features in epilepsy are more closely related to co-occurring emotional symptoms—such as anxiety, difficulties in emotion regulation, and perceived stigma—than to the epilepsy diagnosis itself [27]. When concomitant depression is infrequent and particularly when depressive symptoms are clinically stable, elevated levels of alexithymia may therefore be less prominent.

### Body image

Body image in adolescence is influenced by changes in appearance and social comparison. Some research has also demonstrated increased body dissatisfaction among adolescents with epilepsy, but these findings were not representative of all adolescents [28]. Previous research indicates that body image is likely to be substantially less affected by the diagnosis itself, and far more affected by factors such as weight differences associated with medication, fear of having seizures, and concurrent emotional adversity, including anxiety and social factors [29, 30]. In view of the heterogeneous literature, the lack of group differences identified in our study suggests that adolescents with well-controlled IGE and low comorbid burden may retain similar body image perceptions as their peers.

### Self-esteem

Self-esteem during adolescence is the result of interactions between social relationships, family dynamics, and personal experiences. While several studies have reported decreased self-esteem in adolescents with epilepsy, some data suggest these differences are particularly pronounced among those with concurrent psychiatric conditions, low social support, or difficulty managing the disease [31–33]. Our results, with comparable self-esteem across the epilepsy and control groups, reinforce this broader perspective. In the context of well-controlled IGE, when comorbidities are reduced and clinical care is consistent, epilepsy does not necessarily have a detrimental effect on self-worth.

Beyond clinical stability, participation in meaningful activities and supportive environments may further strengthen self-esteem and well-being. Psychosocial group interventions for young people with epilepsy have been shown to improve confidence and engagement, suggesting that structured support may enhance self-efficacy, social functioning, and quality of life [34].

### Aggressive behavior

Aggressive behavior reflects a range of psychological and environmental influences, and during adolescence, it can occur as a reaction to stress, emotional dysregulation, or social challenges. Although increased aggression has been noted in certain patients with epilepsy, these variations can be correlated with psychiatric comorbidities or with specific antiseizure medications, mainly levetiracetam [35, 36]. Aggression was found to be similarly high in both groups in our sample, indicating that epilepsy as a sole cause is not the most consistent predictor of aggressive behavior. These findings also support the idea that behavioral problems may be more prevalent in the presence of additional risk factors.

The absence of significant group differences in this study appears to be related to the relatively stable clinical characteristics of the adolescents included in the sample. This does not suggest that psychosocial vulnerability is absent in epilepsy in general, but rather that psychosocial outcomes may vary according to seizure control, comorbidity, and contextual factors.

### Limitations

This study has several limitations. A modest sample size may restrict the identification of subtle subgroup differences. The cross-sectional design does not allow conclusions about causality. In addition, psychological scale scores were not stored in a format that permitted analyses according to antiseizure medication type, and the sample size and data distribution limited the use of more advanced statistical approaches, including multivariable regression models.

The relatively low frequency of psychiatric comorbidity in our sample may have influenced the findings, and a more symptomatic group might have produced different results. All participants were recruited from a single center, and psychosocial data were based on self-report instruments, which may limit generalizability. We did not compare outcomes across a wider range of epilepsy subtypes, restricting interpretation according to syndrome characteristics. Studies including more diverse samples may help clarify whether psychosocial functioning differs across epilepsy presentations.

## Conclusions

In this study, no clear differences were observed in body image, self-esteem, aggression, or alexithymia between adolescents with IGE and their healthy peers. In a clinically stable group with well-controlled seizures and limited psychiatric comorbidity, epilepsy itself did not appear to be associated with additional psychosocial burden.

These findings should be interpreted with caution. Larger and more diverse studies are needed to clarify how clinical and contextual factors shape psychosocial outcomes in adolescents with epilepsy.

## Data Availability

The data that support the findings of this study are not publicly available due to ethical and privacy restrictions involving human participants. Anonymized data may be made available from the corresponding author upon reasonable request.

## Abbreviations

BPAQ: Buss-Perry Aggression Questionnaire
CAS: Alexithymia Scale for Children
JAE: Juvenile absence epilepsy
JME: Juvenile myoclonic epilepsy
EGTCSA: Epilepsy with generalized tonic–clonic seizures alone
ADHD: Attention-deficit/hyperactivity disorder

## Code availability

N/A.

## References

[1] Menon RN, Helen Cross J (2025) Childhood epilepsy. Lancet 406(9):636–649. 10.1016/S0140-6736(25)00773-1 40684779 10.1016/S0140-6736(25)00773-1

[2] Wagner J, Bhatia S, Marquis BO, Vetter I, Beatty CW, Garcia R, Joshi C, Kumar G, Rao K, Singhal N, Skjei K (2023) Health disparities in pediatric epilepsy: methods and lessons learned. J Clin Psychol Med Settings 30(2):251–260. 10.1007/s10880-022-09898-1 35930105 10.1007/s10880-022-09898-1 PMC9362655

[3] Austin JK, Dunn DW, Huster GA (2000) Childhood epilepsy and asthma: changes in behavior problems related to gender and change in condition severity. Epilepsia 41(5):615–623. 10.1111/j.1528-1157.2000.tb00217.x 10802769 10.1111/j.1528-1157.2000.tb00217.x

[4] Davies S, Heyman I, Goodman R (2003) A population survey of mental health problems in children with epilepsy. Dev Med Child Neurol 45(5):292–295. 10.1017/s0012162203000550 12729141 10.1017/s0012162203000550

[5] Baca CB, Barry F, Vickrey BG, Caplan R, Berg AT (2017) Social outcomes of young adults with childhood‐onset epilepsy: a case‐sibling‐control study. Epilepsia 58(5):781–791. 10.1111/epi.13726 28378439 10.1111/epi.13726 PMC5429874

[6] Şengül Y, Kurudirek F (2022) Perceived stigma and self-esteem for children with epilepsy. Epilepsy Res 186:107017. 10.1016/j.eplepsyres.2022.107017 36113252 10.1016/j.eplepsyres.2022.107017

[7] Pinquart M, Shen Y (2011) Behavior problems in children and adolescents with chronic physical illness: a meta-analysis. J Pediatr Psychol 36(9):1003–16. 10.1093/jpepsy/jsr042 21810623 10.1093/jpepsy/jsr042

[8] Alessi N, Coleman H, Rayner G (2023) Body image dissatisfaction: a novel predictor of poor quality of life in epilepsy. Epilepsy Behav 141:109149. 10.1016/j.yebeh.2023.109149 36889063 10.1016/j.yebeh.2023.109149

[9] Baiardini I, Abbà S, Ballauri M, Vuillermoz G, Braido F (2011) Alexithymia and chronic diseases: the state of the art. G Ital Med Lav Ergon.;33(1 Suppl A):A47–52. PMID: 21488483. 21488483

[10] Sumer N (2003) Personality and behavioral predictors of traffic accidents: testing a contextual mediated model. Accid Anal Prev 35:949–964 12971930 10.1016/s0001-4575(02)00103-3

[11] Aktas V, Güvenç GB (2006) Relationships between aggressive and prosocial behaviors and age, relational context, and interpersonal sensitivity in female and male adolescents. Hacettepe Üniversitesi Edebiyat Fakültesi Dergisi 23(2):33–264

[12] Onen E (2009) Investigation of the psychometric properties of the aggression scale for Turkish adolescents. Turkish Psychological Counseling and Guidance Journal 4(32):75–84. 10.17066/pdrd.53940

[13] Madran HAD (2012) Validity and reliability study of the Turkish form of the Buss-Perry Aggression Scale. Türk Psikoloji Dergisi 24(2):1–6

[14] Hovardaoğlu S & Özdemir YD (1990) Reliability and validity study of the Body Perception Scale: levels of satisfaction with body images in schizophrenic and major depressive patients. [Unpublished master’s Thesis]. Ankara: Gazi Üniversitesi Sosyal Bilimler Enstitüsü.

[15] Koçak R, Karaboğa M, Baloğlu M (2015) Adaptation of the Alexithymia Scale for children (AAS) to Turkish: a validity and reliability study. Turkish Studies (Electronic) 10(11):1023–1036

[16] Çuhadaroğlu F (1986) Self esteem in adolescents, [Unpublished Master’s Thesis]. Hacettepe University Institute of Social Sciences, Ankara

[17] Mlinar S, Petek D, Cotič Ž, Mencin Čeplak M, Zaletel M (2016) Persons with epilepsy: between social inclusion and marginalisation. Behav Neurol 2016:2018509. 10.1155/2016/2018509 27212802 10.1155/2016/2018509 PMC4861793

[18] Rani A, Thomas PT (2019) Stress and perceived stigma among parents of children with epilepsy. Neurol Sci 40(7):1363–1370. 10.1007/s10072-019-03822-6 30903416 10.1007/s10072-019-03822-6 PMC6579768

[19] Campos-Fernández D, Fonseca E, Olivé-Gadea M, Quintana M, Abraira L, Seijo-Raposo I, Santamarina E, Toledo M (2020) The mediating role of epileptic seizures, irritability, and depression on quality of life in people with epilepsy. Epilepsy Behav 113:107511. 10.1016/j.yebeh.2020.107511 33129044 10.1016/j.yebeh.2020.107511

[20] Operto FF, Pastorino GMG, Pippa F, Padovano C, Vivenzio V, Scuoppo C, Pistola I, Coppola G (2021) Psychiatric symptoms and parental stress in children and adolescents with epilepsy. Front Neurol 12:778410. 10.3389/fneur.2021.778410 34956058 10.3389/fneur.2021.778410 PMC8694379

[21] Kanner AM (2003) The complex epilepsy patient: intricacies of assessment and treatment. Epilepsia 44(Suppl 5):3–8. 10.1046/j.1528-1157.44.s.5.2.x 12859356 10.1046/j.1528-1157.44.s.5.2.x

[22] Khalid B, Waqar Z, Khan S, Ali I, Afzal N, Irfan A, Malik W, Muhammad Adil M, Saddiqa A, Khalil M, Munawar Z (2024) Psychiatric implications of anti-seizure medications in epileptic population. BMC Neurol 24(1):166. 10.1186/s12883-024-03670-8 38773441 10.1186/s12883-024-03670-8 PMC11106872

[23] Gui J, Wang L, Meng L, Zhang X, Ma J, Jiang L (2025) Psychiatric disorders with antiseizure medications in children: an analysis of the FDA adverse event reporting system database. Acta Epileptol 7(1):31. 10.1186/s42494-025-00223-5 40405271 10.1186/s42494-025-00223-5 PMC12100784

[24] Biondi A, Winsor AA, Ebelthite C, Onih J, Pick S, Nicholson TR, Pal DK, Richardson MP (2024) A comprehensive digital mental health screening tool for people with epilepsy: a feasibility study in UK outpatients. Epilepsy Behav 160:110103. 10.1016/j.yebeh.2024.110103 39426050 10.1016/j.yebeh.2024.110103

[25] Keezer MR, Sisodiya SM, Sander JW (2016) Comorbidities of epilepsy: current concepts and future perspectives. Lancet Neurol 15(1):106–15. 10.1016/S1474-4422(15)00225-2 26549780 10.1016/S1474-4422(15)00225-2

[26] Tombini M, Assenza G, Quintiliani L, Ricci L, Lanzone J, Di Lazzaro V (2020) Alexithymia and emotion dysregulation in adult patients with epilepsy. Epilepsy Behav 113:107537. 10.1016/j.yebeh.2020.107537 33242774 10.1016/j.yebeh.2020.107537

[27] Han Y, Zhong R, Yang J, Guo X, Zhang H, Zhang X, Liu Y, Lin W (2023) Alexithymia and related factors among patients with epilepsy. Epilepsy Behav 138:108975. 10.1016/j.yebeh.2022.108975 36399970 10.1016/j.yebeh.2022.108975

[28] Kolstad E, Bjørk M, Gilhus NE, Alfstad K, Clench-Aas J, Lossius M (2017) Young people with epilepsy have an increased risk of eating disorder and poor-quality diet. Epilepsia Open 3(1):40–45. 10.1002/epi4.12089 29588986 10.1002/epi4.12089 PMC5839308

[29] Ferro MA, Ferro AL, Boyle MH (2012) A systematic review of self-concept in adolescents with epilepsy. J Pediatr Psychol 37(9):945–958. 10.1093/jpepsy/jss076 22711910 10.1093/jpepsy/jss076

[30] Lee A, Hamiwka LD, Sherman EM, Wirrell EC (2008) Self-concept in adolescents with epilepsy: biological and social correlates. Pediatr Neurol 38(5):335–339. 10.1016/j.pediatrneurol.2008.01.011 18410849 10.1016/j.pediatrneurol.2008.01.011

[31] Kwong KL, Lam D, Tsui S, Ngan M, Tsang B, Lai TS, Lam SM (2016) Self-esteem in adolescents with epilepsy: psychosocial and seizure-related correlates. Epilepsy Behav 63:118–122. 10.1016/j.yebeh.2016.07.032 27636142 10.1016/j.yebeh.2016.07.032

[32] Mula M, Kanner AM, Jetté N, Sander JW (2021) Psychiatric comorbidities in people with epilepsy. Neurol Clin Pract 11(2):e112–e120. 10.1212/CPJ.0000000000000874 33842079 10.1212/CPJ.0000000000000874 PMC8032418

[33] Temple J, Fisher P, Davies C, Millar C, Gemma Cherry M (2023) Psychosocial factors associated with anxiety and depression in adolescents with epilepsy: a systematic review. Epilepsy Behav 149:109522. 10.1016/j.yebeh.2023.109522 38006843 10.1016/j.yebeh.2023.109522

[34] Dorris L, Broome H, Wilson M, Grant C, Young D, Baker G, Balloo S, Bruce S, Campbell J, Concannon B, Conway N, Cook L, Davis C, Downey B, Evans J, Flower D, Garlovsky J, Kearney S, Lewis S, Stephens V, Turton S, Wright I (2017) A randomized controlled trial of a manual-based psychosocial group intervention for young people with epilepsy [PIE]. Epilepsy Behav 72:89–98. 10.1016/j.yebeh.2017.04.007 28575774 10.1016/j.yebeh.2017.04.007

[35] Brodie MJ, Besag F, Ettinger AB, Mula M, Gobbi G, Comai S, Aldenkamp AP, Steinhoff BJ (2016) Epilepsy, antiepileptic drugs, and aggression: an evidence-based review. Pharmacol Rev 68(3):563–602. 10.1124/pr.115.012021 27255267 10.1124/pr.115.012021 PMC4931873

[36] von Wrede R, Meschede C, Brand F, Helmstaedter C (2021) Levetiracetam, perampanel, and the issue of aggression: a self-report study. Epilepsy Behav 117:107806. 10.1016/j.yebeh.2021.107806 33621813 10.1016/j.yebeh.2021.107806

